# Association of Pharmacist Prescription With Dispensed Duration of Hormonal Contraception

**DOI:** 10.1001/jamanetworkopen.2020.5252

**Published:** 2020-05-20

**Authors:** Maria I. Rodriguez, Alison B. Edelman, Megan Skye, Lorinda Anderson, Blair G. Darney

**Affiliations:** 1 Department of Obstetrics and Gynecology, Oregon Health & Science University, Portland; 2 College of Pharmacy, Oregon State University, Portland

## Abstract

**Importance:**

Since 2016, 11 states have expanded the scope of pharmacists to include direct prescription of hormonal contraception. Dispensing greater than 1 month’s supply is associated with improved contraceptive continuation rates and fewer breaks in coverage. Scant data exist on the practice of pharmacist prescription of contraception and its outcomes compared with traditional, clinic-based prescriptions.

**Objective:**

To compare the amount of hormonal contraceptive supply dispensed between pharmacists and clinic-based prescriptions. Prescribing patterns were assessed by describing prescribing practices for women with contraindications to combined hormonal contraception. Characteristics of women seeking hormonal contraception directly from pharmacists were also described.

**Design, Setting, and Participants:**

This cohort study surveyed women aged 18 to 50 years who presented to pharmacies in California, Colorado, Hawaii, and Oregon for hormonal contraception prescribed by a clinician or a pharmacist between January 30 and November 1, 2019.

**Exposures:**

Pharmacist or clinic-based prescription of contraception.

**Main Outcomes and Measures:**

Months of contraceptive supply dispensed.

**Results:**

Four hundred ten women (mean [SD] age, 27.1 [7.7] years) were recruited who obtained contraception directly from a pharmacist (n = 144) or by traditional clinician prescription (n = 266). Women obtaining contraception from a pharmacist were significantly younger (82 [56.9%] vs 115 [43.2%] participants aged 18-24 years; *P* = .03), had less education (38 [26.4%] vs 100 [37.6%] with a bachelor degree; *P* = .002), and were more likely to be uninsured (16 [11.1%] vs 8 [3.0%] participants; *P* = .001) compared with women with a prescription from a clinician. Pharmacists were significantly more likely to prescribe a 6-month or greater supply of contraceptives than clinicians (6.9% vs 1.5%, *P* < .001) and significantly less likely to only prescribe a 1-month supply (42 [29.2%] vs 118 [44.4%] prescriptions; *P* < .001). Controlling for all covariates, women seen by pharmacists had higher odds of receipt of a 6-month or greater supply of contraceptives compared with those seen by clinicians (odds ratio = 3.55; 95% CI, 1.88-6.70). Pharmacists were as likely as clinicians to prescribe a progestin-only method to women with a potential contraindication to estrogen (n = 60 women; 8 [20.0%] vs 6 [30.0%], *P* = .52).

**Conclusions and Relevance:**

These findings suggest that pharmacist prescription of contraception may be associated with improved contraceptive continuation by preventing breaks in coverage through the provision of a greater supply of medication. Efforts are needed to educate prescribing providers on the importance of dispensing 6 months or greater contraceptive supply.

## Key Points

**Question:**

Is pharmacist prescription of contraception associated with dispensing a longer duration of contraceptive coverage?

**Findings:**

In this cohort study of 410 women in 4 US states, we found that pharmacist prescription of hormonal contraception was associated with a contraceptive supply of more than 6 months dispensed compared with traditional clinic-based prescriptions. Women receiving contraception from pharmacists were younger, had less education, and were more likely to be uninsured than women seeing clinicians.

**Meaning:**

These findings suggest that pharmacist prescription of contraception is a promising strategy to promote consistent use of hormonal contraception.

## Teaser

This cohort study evaluates the association of pharmacist vs clinician prescription with the dispensed duration of hormonal contraception among women in 4 US states.

## Introduction

Reducing the proportion of unintended pregnancies is a national public health priority in the United States.^1^ The US unintended pregnancy rate has fallen in recent decades, but 45% of all pregnancies are still unintended.^2^ Contraception is highly effective at preventing unintended pregnancy, but barriers exist to effective and consistent use. Innovations in service delivery, including task sharing, are needed to improve access to and continuation of contraception, especially among vulnerable populations.^3^

Pharmacist prescription of contraception is a promising strategy to increase access to contraception for new users or to promote continuation among current users.^4,5^ Pharmacies are generally located in the community they serve, have extended hours compared with clinics, and do not require an appointment to be seen.^4^ It is believed that these characteristics will reduce breaks in contraceptive supply for current users and streamline access for women wishing to initiate a method.

In 2016, Oregon became the first state to implement legislation allowing pharmacists to independently prescribe hormonal contraception, including the pill, patch, or ring, directly to the patient without a traditional clinic visit.^6^ Pharmacists voluntarily elect to complete additional training and follow a prescribing algorithm. This algorithm is based on the Centers for Disease Control and Prevention’s Medical Eligibility for Contraceptive Use, and its use is designed to aid providers in safely prescribing contraception.^7,8,9^ Since the implementation of this policy in Oregon, 10 other states have passed legislation to allow pharmacist prescription of hormonal contraception without oversight from a supervising clinician.^10,11^ This rapid, national expansion of a new cadre of contraceptive prescribers has key public health implications. It is important to understand who pharmacists are reaching and how their prescribing patterns are similar to, or distinct from, clinicians.

However, existing data on who is utilizing pharmacist prescription of contraception in the US, and why, are limited.^4,12,13,14^ Preliminary evidence from 2 states suggest that the majority of women accessing contraception in a retail pharmacy are insured and using the contraceptive pill.^12^ Reports conflict as to whether the majority of women are new users of contraception or continuing a method.^4,12,14^ Data on pharmacists’ contraceptive prescribing practices are similarly limited.^15,16^ Previous research has established that dispensing a larger contraceptive supply is associated with improved contraceptive continuation rates and a reduction in unintended pregnancy.^17,18,19^ Contraceptive supply dispensed is determined by the amount prescribed, insurance companies’ policies on reimbursement, and the individual’s preference. Whether pharmacists supply a similar quantity of months of contraceptive supply as clinicians when they prescribe hormonal contraception is unknown to date.

The purpose of this study was to test whether there are differences in the amount of contraceptive supply dispensed by pharmacists compared with clinicians.

## Methods

We used the baseline wave of a prospective cohort study of women presenting for contraception in pharmacies across 4 states in this analysis.^20^ The full study is powered to detect a 10% difference in contraceptive continuation rates at 1 year.^5,20^All analyses and reporting of results were conducted in accordance with the Strengthening the Reporting of Observational Studies in Epidemiology (STROBE) reporting guideline for cohort studies.^21^ The institutional review board at Oregon Health & Science University approved the study protocol, and all participants provided written informed consent.

### Study Population

Women presenting at participating pharmacies in California, Hawaii, Colorado, and Oregon for contraception between January 30 and November 1, 2019, were eligible for enrollment (eFigure 1 in the Supplement). We recruited at a total of 139 pharmacies (California, 46; Colorado, 14; Hawaii, 10; and Oregon, 69). Pharmacies were retail, chain pharmacies or associated with a university health center. A summary of each state’s approach to pharmacist prescription of contraception is provided in the eTable in the Supplement. We recruited women aged 18 to 50 years who received at least 1 month of hormonal contraception (pill, patch, ring, or injectable) from a clinician or pharmacist prescription. Only individuals who provided the method of contraception and number of months of contraception they received were included in the study. Participants indicating they were using the method for a medical reason (eg, dysmenorrhea or menorrhagia) or that they had a concomitant permanent form of contraception were excluded. Pharmacy staff at these locations were trained to provide eligible women with sealed recruitment packets after they dispensed their contraception. Participants had the option of completing a paper version of the consent form and survey provided in the packet or following a link to an online version of the consent form and survey. Written informed consent was obtained from all participants. eFigure 2 in the Supplement provides a summary of our recruitment process. Survey data were managed using REDCap electronic data capture tools hosted at Oregon Health & Science University.^22^

### Variables

Our key independent variable was prescribing provider type: clinician or pharmacist. Our primary outcome was months of hormonal contraception dispensed. Previous research has focused on a range of time periods, between 3 and 13 months of supply dispensed. Recent data indicate that the majority of women (70%) receive a supply of 3 months or less, and 15% receive 6 months or more.^23^ We therefore examined contraceptive supply as a dichotomous variable (≥6-month supply vs <6-month supply).^24,25,26,27^

Our secondary outcome was the proportion of prescriptions containing estrogen for individuals with a potential contraindication to a combined method. We asked individuals if they had health conditions or behaviors that indicated a potential contraindication to hormonal contraceptive use.^28^ We utilized the wording and checklist approach validated in previous studies to elicit information on potential contraindications to estrogen.^29,30^ Health conditions classified as category 3 or 4 by the Centers for Disease Control and Prevention’s Medical Eligibility Criteria for contraceptive use were used to define a potential contraindication.^28^ Women receiving estrogen-containing methods were flagged as having a potential contraindication to combined hormonal contraception if they had history of migraines with aura, history of blood clots, or hypertension or were over 40 years of age with hypertension or current tobacco use (multiple risk factors for atherosclerotic cardiovascular disease).^7^

We collected demographic, health history, pregnancy intention, and preference information from each study participant. Demographic data included self-reported age, race/ethnicity, ZIP code of residence, level of education, insurance, relationship status, and employment status. Age was recorded in years and collapsed into a dichotomous variable for regression analyses based on the median age of the sample (age under 26 years or 26 years and older). Self-reported race/ethnicity was captured using the standard National Institutes of Health categories. We used the participant’s ZIP code to determine urban or rural residence, using guidance from the Federal Office of Rural Health Policy.^31^ Education was classified as a categorical variable with options ranging from no schooling to completion of a doctoral degree. Women were asked if they had health insurance (yes or no). We classified relationship status as single, never married; married or partnered; widowed; divorced; or separated.

We specifically sought to elicit information about pregnancy intention, as individuals planning pregnancy within the year may request that fewer months of contraception be dispensed. Women were asked if they planned a pregnancy within the next year (yes, no, or unsure). Women answering no or unsure were asked how important it was to them to avoid a pregnancy (extremely important, important, or open to pregnancy).^32^ We asked whether women had previously used the method they were prescribed at time of enrollment, as it is possible that repeat users may be more likely to request that a larger supply be dispensed.

### Statistical Analysis

We used descriptive statistics and bivariate tests (χ^2^ or Fisher exact for small cell sizes) of association to characterize the sample by provider type (pharmacist vs clinician) and outcome (6-month supply dispensed). We described number of months of contraceptive supply dispensed and the presence of potential contraindications to estrogen by provider type. We developed a multivariable logistic regression model to evaluate the association between provider type and our outcome (greater than or less than a 6-month supply dispensed). Model covariates included age, insurance status, race/ethnicity, rural residence, and state of residence. We included state to control for any unmeasured differences in dispensing practices by state. We selected these covariates a priori based on factors associated with method discontinuation and unintended pregnancy.^2,33,34,35^ We chose not to include previous use of the method and pregnancy intention, because these were not different by provider type or outcome in bivariate analyses.

We examined missingness in our data. We examined demographic characteristics by outcome missingness, and there were no significant differences between those with and without outcome data. All demographic variables were missing under 1% of responses. We conducted several sensitivity analyses. First, we ran models that included previous use of the method and pregnancy intention variables. We also ran a model restricted to the subsample of insured women (n = 385). Finally, we ran models making assumptions about outcomes among those missing outcome data. All results were robust to these changes, and we include only our final model. All analyses were performed using Stata 16.0 (StataCorp LP). *P* < .05, using a 2-sided test, indicated statistical significance. Data were analyzed from October 1 to November 25, 2019.

## Results

Our sample included 410 women (mean [SD] age, 27.1 [7.7] years) presenting for hormonal contraception (response rate, 50.8%) (eFigure 2 in the Supplement). A larger proportion of women received a prescription from a clinician (266 [64.9%]) than a pharmacist (144 [35.1]%) (Table 1). Women receiving contraception from a pharmacist tended to have a lower level of education and were slightly younger; 82 pharmacist prescriptions (56.9%) were to women aged 18 to 24 years vs 115 clinician prescriptions (43.2%) (*P* = .03), and 38 women (26.4%) with a pharmacist prescription had a bachelor degree vs 100 women (37.6%) with a clinician prescription (*P* = .002) (Table 1). A greater proportion of women seeking contraception from a pharmacist (16 [11.1%]) than from a clinician (8 [3.0%]) reported being uninsured (*P* = .001). A large majority of women in both groups reported that they did not plan pregnancy within the year, and that avoiding pregnancy was “extremely important” to them (187 women with a clinician prescription [70.3%] vs 115 with a pharmacist prescription [79.9%]) (Table 1).

**Table 1.** Participant Demographic Characteristics by Type of Contraceptive Prescriber

**C**haracteristic	No. (%)^a^			*P* value
	Clinician (n = 266)	Pharmacist (n = 144)	Total (N = 410)	
Age, y				
18-24	115 (43.2)	82 (56.9)	197 (48.0)	.03
25-29	62 (23.3)	26 (18.1)	88 (21.5)	
30-34	42 (15.8)	22 (15.3)	64 (15.6)	
≥35	47 (17.7)	14 (9.7)	61 (14.9)	
Race/ethnicity				
White	193 (72.6)	95 (66.0)	288 (70.2)	.21^b^
Hispanic or Latina	15 (5.6%)	9 (6.3)	24 (5.9)	
Black	1 (0.4)	2 (1.4)	3 (0.7)	
Asian	24 (9.0)	17 (11.8)	41 (10.0)	
American Indian or Alaska Native	0	1 (0.7)	1 (0.2)	
Native Hawaiian or Pacific Islander	0	2 (1.4)	2 (0.5)	
Multiracial	33 (12.4)	18 (12.5)	51 (12.4)	
Educational level				
Less than high school	10 (3.8)	1 (0.7)	11 (2.7)	.002^b^
High school diploma or GED	26 (9.8)	25 (17.4)	51 (12.4)	
Some college	79 (29.7)	61 (42.4)	140 (34.1)	
Bachelor degree	100 (37.6)	38 (26.4)	138 (33.7)	
Master, professional, or doctoral degree	51 (19.2)	19 (13.2)	70 (17.1)	
Single (vs partnered)	190 (71.4)	108 (75.0)	298 (72.7)	.37
Rural residence (vs urban)	27 (10.2)	14 (9.7)	41 (10.0)	.89
State				
California	46 (17.3)	25 (17.4)	71 (17.3)	.66^b^
Colorado	42 (15.8)	23 (16.0)	65 (15.9)	
Hawaii	7 (2.6)	1 (0.7)	8 (2.0)	
Oregon	171 (64.3)	95 (66.0)	266 (64.9)	
Uninsured	8 (3.0)	16 (11.1)	24 (5.9)	.001
History of pregnancy	56 (21.1)	20 (13.9)	76 (18.5)	.07
Openness to pregnancy in next 12 mo^c^				
Extremely important to avoid pregnancy	187 (72.5)	115 (80.4)	302 (75.3)	.14
Important to avoid pregnancy	31 (12.0)	17 (11.9)	48 (12.0)	
Open to pregnancy if it happens	21 (8.1)	7 (4.9)	28 (7.0)	
Would like to be pregnant	19 (7.4)	4 (2.8)	23 (5.7)	
Experienced user of hormonal contraception	227 (85.3)	123 (85.4)	350 (85.4)	.98

Abbreviation: GED, General Educational Development.

^a^ Individual variable denominators differ depending on missingness.

^b^ *P* value based on the Fisher exact test.

^c^ Four hundred one participants reported being open to pregnancy in the next 12 months (258 with clinician prescriptions and 143 with pharmacist prescriptions).

Overall, 52 (12.7%) women received a 6-month or greater supply of contraceptives (Table 2). Individuals receiving a 6-month supply were more likely to be younger (aged 18-24 y: 38 [73.1%] vs 159 [44.4%]; *P* = .001), to be single (44 [84.6%] vs 254 [71.1%]; *P* = .04), to have never been pregnant (2 [3.9%] vs 74 [20.7%]; *P* = .003), and to reside in Oregon (44 [84.6%] vs 222 [62.0%]; *P* = .003).The number of months of contraceptive supply dispensed varied markedly by type of prescribing provider. Pharmacists were significantly more likely to prescribe a 6-month or greater supply of contraceptives than were clinicians (10 [6.9%] vs 4 [1.5%] prescriptions; *P* < .001) and significantly less likely to only prescribe a 1-month supply (42 [29.2%] vs 118 [44.4%] prescriptions; *P* < .001) (Table 3). Overall, we found that the majority of providers were dispensing less than 3 months of coverage (126 clinicians [85.0%] and 100 pharmacists [69.5%]). Because of incomplete responses from participants, 6.1% of observations on our primary outcome, months of contraceptive coverage dispensed, were missing.

**Table 2.** Participant Demographics by Months of Contraception Dispensed

Characteristic	No. (%)^a^			*P* value
	<6 mo (n = 358)	≥6 mo (n = 52)	Total (n = 410)	
Age, y				
18-24	159 (44.4)	38 (73.1)	197 (48.0)	<.001
25-29	83 (23.2)	5 (9.6)	88 (21.5)	
30-34	59 (16.5)	5 (9.6)	64 (15.6)	
≥35	57 (15.9)	4 (7.7)	61 (14.9)	
Race/ethnicity				
White	247 (69.0)	41 (78.8)	288 (70.2)	.46^b^
Hispanic or Latina	23 (6.4)	1 (1.9)	24 (5.9)	
Black	3 (0.8)	0	3 (0.7)	
Asian	39 (10.9)	2 (3.8)	41 (10.0)	
American Indian or Alaska Native	1 (0.3)	0	1 (0.2)	
Native Hawaiian or Pacific Islander	2 (0.6)	0	2 (0.5)	
Multiracial	43 (12.0)	8 (15.4)	51 (12.4)	
Educational level				
Less than high school	11 (3.1)	0	11 (2.7)	.10^b^
High school diploma or GED	44 (12.3)	7 (13.5)	51 (12.4)	
Some college	114 (31.8)	26 (50.0)	140 (34.1)	
Bachelor degree	126 (35.2)	12 (23.1)	138 (33.7)	
Master, professional, or doctoral degree	63 (17.6)	7 (13.5)	70 (17.1)	
Single (vs partnered)^c^	254 (71.1)	44 (84.6)	298 (72.9)	.04
Rural residence (vs urban)	27 (7.5)	14 (26.9)	41 (10.0)	.92
State				
California	67 (18.7)	4 (7.7)	71 (17.3)	.02^b^
Colorado	61 (17.0)	4 (7.7)	65 (15.9)	
Hawaii	8 (2.2)	0	8 (2.0)	
Oregon	222 (62.0)	44 (84.6)	266 (64.9)	
Uninsured	22 (6.1)	2 (3.8)	24 (5.9)	.75^b^
History of pregnancy	74 (20.7)	2 (3.8)	76 (18.5)	.01
Openness to pregnancy in next 12 mo^d^				
Extremely important to avoid pregnancy	261 (74.8)	41 (78.8)	302 (75.3)	.68^b^
Important to avoid pregnancy	41 (11.7)	7 (13.5)	48 (12.0)	
Open to pregnancy if it happens	25 (7.2)	3 (5.8)	28 (7.0)	
Would like to be pregnant	22 (6.3)	1 (1.9)	23 (5.7)	
Experienced user of hormonal contraception	304 (84.9)	46 (88.5)	350 (85.4)	.50

Abbreviation: GED, General Educational Development.

^a^ Individual variable denominators differ depending on missingness.

^b^ *P* value based on Fisher exact test.

^c^ Four hundred nine participants reported on their relationship status (357 with <6 months dispensed and 52 with ≥6 months dispensed).

^d^ Four hundred one participants reported on their oppenness to pregnancy in the next 12 months (349 with clinician prescriptions and 52 with pharmacist prescriptions).

**Table 3.** Amount of Contraception Dispensed by type of Contraceptive Prescriber

Months of medication, No.	No. (%)			*P* value
	Clinician (n = 266)	Pharmacist (n = 144)	Total (n = 410)	
1	118 (44.4)	42 (29.2)	160 (39.0)	<.001
2-3	108 (40.6)	58 (40.3)	166 (40.5)	
4-5	20 (7.5)	12 (8.3)	32 (7.8)	
6-11	4 (1.5)	10 (6.9)	14 (3.4)	
12	16 (6.0)	22 (15.3)	38 (9.3)	

In our multivariable regression model, a pharmacist prescriber was significantly associated with dispensing a 6-month or greater supply of hormonal contraceptives (odds ratio [OR] = 3.55; 95% CI, 1.86-6.7), controlling for all covariates (Table 4). Younger age (≤25 y; OR, 2.09; 95% CI, 1.06-4.13) and state (Oregon; OR, 3.06; 95% CI, 1.36-6.88) were also positively associated with receipt of 6 or more months of hormonal contraception (Table 4).

**Table 4.** Association Between Provider Type and Receipt of 6 or More Months of Contraceptive Supply Dispensed

Variable	Odds Ratio (95% CI)	*P* value
Pharmacist prescription	3.55 (1.88-6.70)	<.001
Patient aged ≤25 y	2.09 (1.06-4.13)	.03
Nonwhite race/ethnicity	0.56 (0.27-1.18)	.13
Rural residence	0.92 (0.33-2.60)	.88
Oregon state prescription	3.06 (1.36-6.88)	.01
Uninsured	0.47 (0.10-2.22)	.34

We also examined how differences in prescribing to individuals with a potential contraindication to estrogen may vary by provider type. Overall, the number of women with potential contraindications to estrogen was low and similar to what has been reported previously in the literature (13.9%; n = 60).^36,37^ Overall, we found that 43 women (71.7%) with a potential contraindication to estrogen received a combined method (Table 5). There was no significant difference between pharmacists and clinicians in prescription of a progestin-only method of contraception among women with a potential contraindication to estrogen (8 [20.0%] vs 6 [30.0%] prescriptions; *P* = .52) (Table 5).

**Table 5.** Potential Contraindications by Type of Prescription Provider

Variable	No. (%)			*P* value
	Clinician (n = 266)	Pharmacist (n = 144)	Total (n = 410)	
Potential contraindication with estrogen				
High blood pressure	7 (2.6)	5 (3.5)	12 (2.9)	.46^a^
History of blood clots	2 (0.8)	0	2 (0.5)	.54^a^
Migraines with auras	32 (12.0)	15 (10.4)	47 (11.5)	.62
Current smoker and age >35 y	1 (0.4)	1 (0.7)	2 (0.5)	>.99
Multiple risk factors for atherosclerotic cardiovascular disease^b^	2 (0.8)	4 (2.8)	6 (1.5)	.19^a^
Women with a potential contraindication^c^	40 (15.0)	20 (13.9)	60 (14.6)	.75
Among women with a potential contraindication (n = 60)^c^				
Prescribed a method containing estrogen	32 (80.0)	14 (70.0)	43 (71.7)	.52^a^
Prescribed a progestin-only method	8 (20.0)	6 (30.0)	14 (23.3)	

^a^ *P* value based on Fisher method on 2 × 2 cross tabulation (Fisher exact test).

^b^ Defined as being of age ≥40 y and either being a current smoker or having high blood pressure.

^c^ Defined as having high blood pressure, a history of blood clots, or migraines with auras; being a current smoker >35 y of age; or having multiple risk factors for atherosclerotic cardiovascular disease.

## Discussion

In this cohort study, we found that women obtaining a prescription from a pharmacist received a greater supply of contraception, which has been associated with higher rates of contraceptive continuation and adherence.^18,19,25^ Women receiving contraception from pharmacists were more likely to be younger, have less education, and be uninsured, which are known risk factors for unintended pregnancy.^38^

We found that pharmacists were significantly more likely to dispense 6 months or greater of contraceptives, a strategy that has proven to be a cost-effective intervention to improve contraceptive continuation and prevent unintended pregnancy.^18,19,39,40^ A large cohort study from California demonstrated that women who were prescribed a 12-month supply of contraceptives were nearly twice as likely to be continuing to use the method at 15 months than women who received only a 1-month supply (40% vs 21%).^18^ These findings were validated by a randomized trial demonstrating that a 7- vs 3-month supply dispensed led to a significant increase in continuation rates at 6 months.^25^ This improvement in continuation rates is associated with a reduction in unintended pregnancy. Women receiving a 1-year supply of contraceptives have a 30% reduction in the odds of having an unplanned pregnancy when compared with women who are dispensed less than 3 months of coverage.^18^

Local governments have recognized the importance of the quantity of contraceptive supply dispensed on preventing unintended pregnancy; 17 states and the District of Colombia have passed laws mandating insurance coverage for dispensing a 1-year supply of contraceptives at a time.^41^ However, scant data exist to date on whether these policies have been fully implemented (ie, accepted by insurance companies and practice changes incorporated by providers). It is believed that these policies have not been widely implemented, owing to exceptions for self-insured private plans and lack of outreach to providers about the coverage change.^23,42,43^ Data on how policies mandating 12-month coverage of contraception have been implemented are lacking.

We identified a need to educate all contraceptive providers on the importance of the quantity of contraceptive supply dispensed. Although our study found that pharmacists were more likely to prescribe a supply of 6 months or greater, we also found that a majority of providers were dispensing less than 3 months of coverage (85.0% of clinicians and 69.5% of pharmacists). In Colorado’s and Oregon’s protocols for pharmacist prescription of contraception, pharmacists are encouraged to prescribe and dispense up to a 12-month supply on the basis of professional judgment and patient preference.^44,45^ A similar prompt in clinical electronic health records may increase the supply dispensed by clinicians.

Combined hormonal contraception has an excellent safety profile.^46,47^ Professional and public health organizations have advocated that it be available over the counter without regulation.^46^ Removing the prescription barrier to hormonal contraception requires a change in US Food and Drug Administration status, a costly and time-consuming process. Pharmacist prescription of contraception is increasingly common nationally as states seek innovative solutions to address the acute unmet need for contraception that exists across the US.^11^ Opponents of removing the prescription requirement for hormonal contraception have cited safety as a concern.

Our study found that the overall prevalence of individuals seeking contraception with potential contraindications to estrogen was low and consistent with what has previously been reported in the literature.^36,37^ We also found that pharmacists are as likely as clinicians to prescribe a safer, progestin-only method for women with a potential contraindication to estrogen. Although there was no difference by provider type, our analysis was underpowered to study this outcome. We did still find that a meaningful proportion (71.7%) of women with potential contraindications received an estrogen-containing method across provider type. This is a higher rate than what has previously been reported in the literature.^36,37^ We believe that it is unlikely that a woman with a true absolute contraindication to estrogen received an estrogen-containing method from either provider type for the following reasons. Our assessment of safety was conservative, as we defined potential contraindications as a Medical Eligibility Criteria category 3 or 4. Many women can still safely use an estrogen-containing contraceptive method who have a category 3 condition. Women are also known to be more conservative on self-assessment of potential contraindications to contraception than providers.^29^ A provider may have reviewed the potential contraindication and appropriately determined the condition not to be a contraindication. We did not capture this possibility in our survey as it was the woman and not the provider completing it. Overall, the use of checklists by providers appears to improve safety. In states, such as California, Colorado, and Oregon, where a checklist is used as part of the pharmacist prescribing process, we believe that a sufficiently powered study might demonstrate an improved safety profile with pharmacist prescription of combined hormonal contraception. A study powered to examine differences in prescription of contraception to women with potential contraindications to estrogen is needed.

Our study addresses an important gap in the literature by providing information about an increasingly common change to health care delivery: direct prescription by pharmacists.^11^ Our study represents, to date, the largest cohort of women across multiple states in the US who have received contraception from a pharmacist. Our findings on who is seeking contraceptive care in the pharmacy and their experiences with doing so provides novel and needed information on how we can strengthen contraceptive services in both pharmacies and clinics.

### Limitations

Our study is not without limitations and should be interpreted with these factors in mind. The majority of women receiving prescriptions from a pharmacists obtained their contraceptive in Oregon. This affects the generalizability of our results. Each state has a distinct approach to pharmacist prescription of contraception in terms of the training required, the methods covered, and insurance reimbursement (eTable in the Supplement). Our study was not powered to detect how differences in states policies affect contraceptive utilization. We also lack data on women’s type of insurance, which may affect the supply dispensed. Although all of the states we recruited in have policies around insurance coverage for a 12-month supply of contraceptives, it is not known whether these policies are fully implemented. We found that pharmacists were seeing a larger proportion of uninsured women than were clinicians, although the proportion of uninsured women in our sample was small, limiting our ability to make an inference about the association between insurance, provider type, and supply dispensed. Consistent with previous studies, our data on potential contraindications to estrogen were self-reported and thus subject to recall bias. We used validated checklists to assess for medical contraindications.^29^ We are also evaluating pharmacy-direct prescribing relatively early in its implementation; future studies may see changes in who is accessing services and why. We were unable to analyze for differences by type of pharmacy in our study, although we did examine differences by state. In addition, we were missing 6.1% of observations on our primary outcome, months of contraceptive coverage; however, we compared women missing and not missing outcome data and found no differences on other demographic covariates, which suggests that missing outcome data did not introduce bias.

## Conclusions

Our findings are promising that pharmacist prescription of contraception safely promotes contraceptive continuation through minimizing breaks in contraceptive coverage by dispensing a supply of greater than 6 months. Longitudinal data are needed to confirm whether this finding translates to improved contraceptive continuation and reduced rates of unintended pregnancy among this population. As pharmacist prescription of contraception continues to increase nationally, evidence on this practice is needed to guide care. Our study supports a growing body of evidence demonstrating that pharmacist prescription of contraception is a promising strategy to improve contraceptive access.^4,5,13^

## Notes

Supplement. **eFigure 1.** Urban and Rural Census Tracts Where Participants Received Prescriptions From Pharmacists **eFigure 2.** Study Enrollment Flow Chart **eTable.** Summary of State Programs
