# Supplementary material for: Association of Pharmacist Prescription With Dispensed Duration of Hormonal Contraception
Source: JAMA Netw Open. 2020 May 20;3(5):e205252. doi: 10.1001/jamanetworkopen.2020.5252 (PMC7240351; doi:10.1001/jamanetworkopen.2020.5252)
Supplement: Supplement. — eFigure 1. Urban and Rural Census Tracts Where Participants Received Prescriptions From Pharmacists eFigure 2. Study Enrollment Flow Chart eTable. Summary of State Programs [file jamanetwopen-3-e205252-s001.pdf]

Supplementary Online Content

Rodriguez MI, Edelman AB, Skye M, Anderson L, Darney BG. Association of pharmacist prescription with dispensed duration of hormonal contraception. *JAMA Netw Open*. 2020;3(5):e205252. doi:10.1001/jamanetworkopen.2020.5252

eFigure 1. Urban and Rural Census Tracts Where Participants Received Prescriptions From Pharmacists

eFigure 2. Study Enrollment Flow Chart

eTable. Summary of State Programs

This supplementary material has been provided by the authors to give readers additional information about their work.

© 2020 Rodriguez MI et al. *JAMA Network Open.*

eFigure 1: Urban and rural census tracts where participants received prescriptions from pharmacists

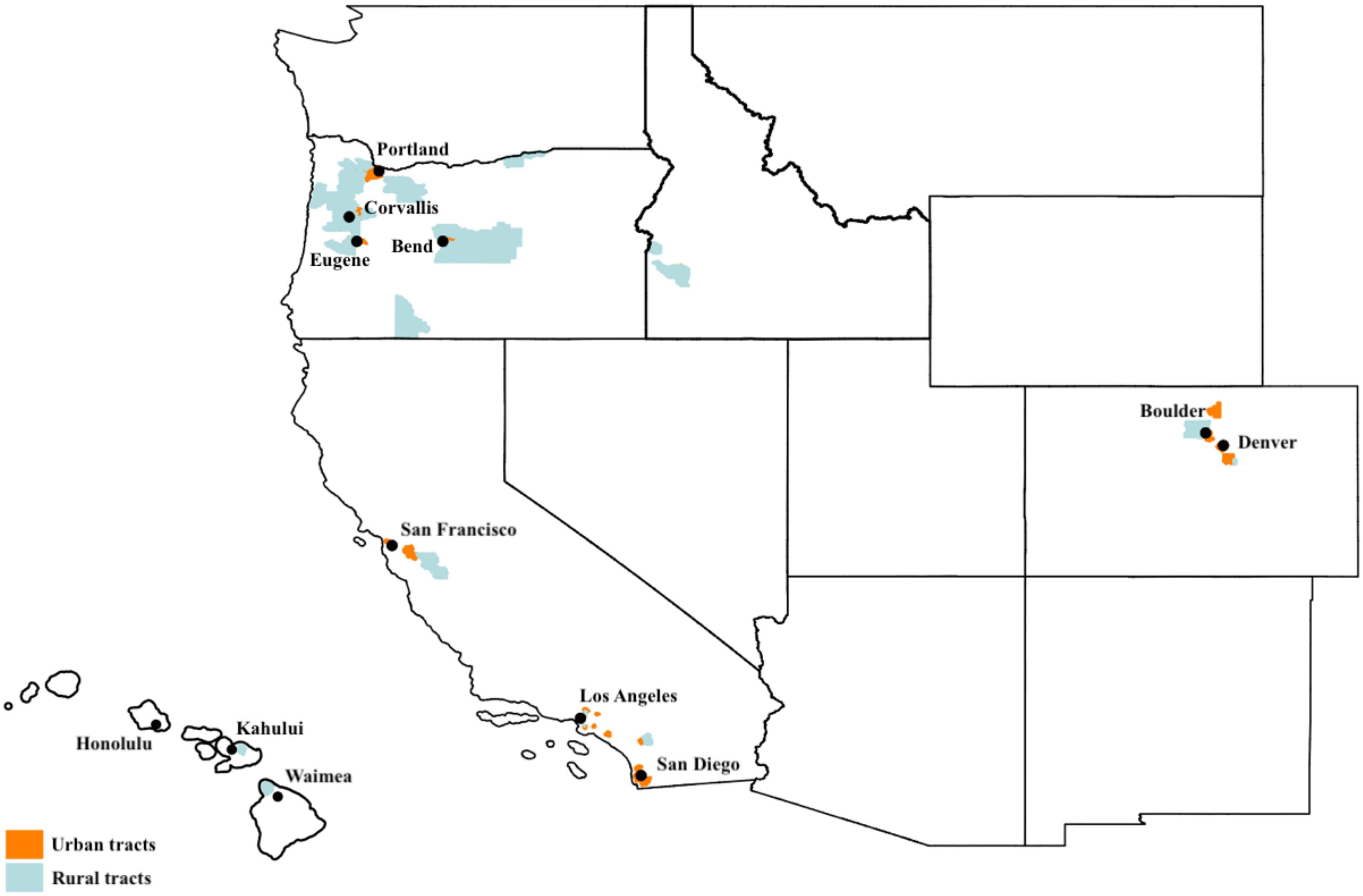

© 2020 Rodriguez MI et al. *JAMA Network Open.*

eFigure 2: Study enrollment flow chart

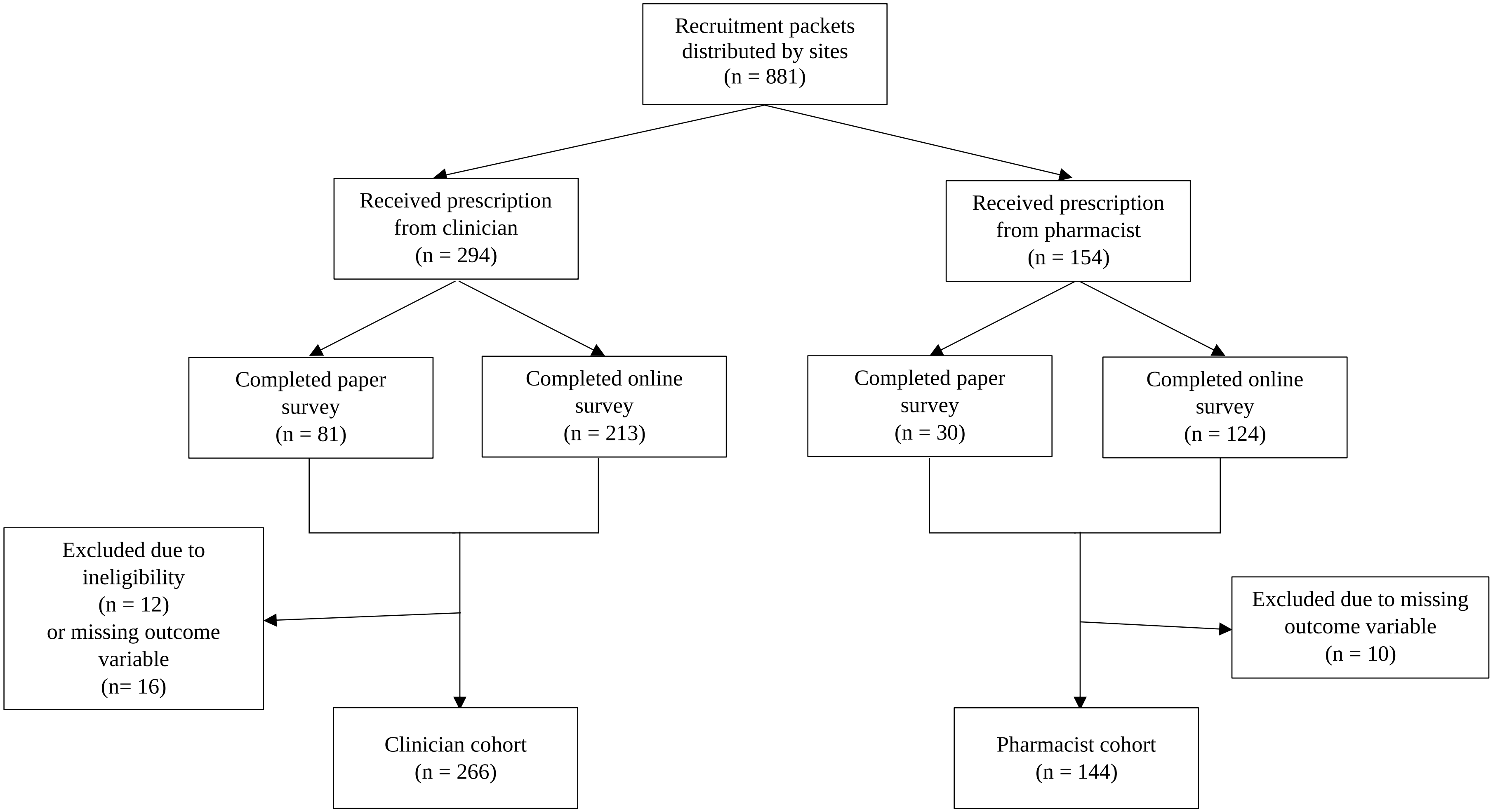

© 2020 Rodriguez MI et al. *JAMA Network Open.*

eTable: Summary of state programs

State	Implementation Date	Methods Included	Prescribing Authority	Training Requirement	Age Restrictions	Reimbursement Practices
Oregon	Jan. 2016	-2016-17: Pill & Patch<br>-2017-Present: Pill, Patch, Ring, Injection	Unrestricted Category-Specific*	Board of Pharmacy approval and ACPE-accredited	$\geq$ 18 y.o. or previous prescription (sunsets in Jan. 2020)	Medicaid and some 3rd-party coverage (started in Jan. 2016)
California	April 2016	Pill, Patch, Ring, Injection	State Protocol**	1 hour related to self-administered contraception, or graduated after 2014	None	Medicaid (started in April 2019)
Colorado	Feb. 2017	Pill & Patch	State Protocol**	ACPE-accredited related to pharmacist's prescribing contraception	$\geq$ 18 y.o.	None
Hawaii	July 2017	Pill, Patch, Ring	Unrestricted Category-Specific*	ACPE-accredited related to pharmacist's prescribing contraception every 2 years	None	None

ACPE: Accreditation Council for Pharmacy Education

Types of Prescribing authority being utilized in various states[1]:

***Unrestricted category-specific:** allows pharmacist's to autonomously prescribe within a specified category of medications and for a medical purpose (often to benefit public health). This is done without entering into a collaborative agreement with a supervising physician.

****State-wide protocol:** allows pharmacist's to autonomously prescribe when they follow and meet qualifying criteria set out in a protocol published by an empowered state body. When pharmacist's follow the state protocol a collaborative agreement with a supervising physician is not needed.

© 2020 Rodriguez MI et al. *JAMA Network Open.*
